# Immune Enhancement Effects of Neutral Lipids, Glycolipids, Phospholipids from *Halocynthia aurantium* Tunic on RAW264.7 Macrophages

**DOI:** 10.4014/jmb.2307.07003

**Published:** 2023-11-15

**Authors:** A-yeong Jang, Weerawan Rod-in, Il-shik Shin, Woo Jung Park

**Affiliations:** 1 Department of Wellness-Bio Industry, Gangneung-Wonju National University, Gangneung, Gangwon 25457, Republic of Korea; 2 Department of Marine Bio Food Science, Gangneung-Wonju National University, Gangneung, Gangwon 25457, Republic of Korea; 3 Department of Agricultural Science, Faculty of Agriculture Natural Resources and Environment, Naresuan University, Phitsanulok 65000 Thailand

**Keywords:** *Halocynthia aurantium*, tunic, lipids, macrophages

## Abstract

Fractionated lipids of *Halocynthia aurantium* (Pyuridae) have been demonstrated to possess anti-inflammatory properties. However, their modulatory properties have not been reported yet. Thus, the objective of this study was to determine immune enhancing effects of fractionated lipids from *H. aurantium* tunic on macrophage cells. The tunic of *H. aurantium* was used to isolate total lipids, which were then subsequently separated into neutral lipids, glycolipids, and phospholipids. RAW264.7 cells were stimulated with different concentrations (0.5, 1.0, 2.0, and 4.0%) of each fractionated lipid. Cytotoxicity, production of NO, expression levels of immune-associated genes, and signaling pathways were then determined. Neutral lipids and glycolipids significantly stimulated NO and PGE_2_ production and expression levels of *IL-1β*, *IL-6*, *TNF-α*, and *COX-2* in a dose-dependent manner, while phospholipids ineffectively induced NO production and mRNA expression. Furthermore, it was found that both neutral lipids and glycolipids increased NF-κB p-65, p38, ERK1/2, and JNK phosphorylation, suggesting that these lipids might enhance immunity by activating NF-κB and MAPK signaling pathways. In addition, *H. aurantium* lipids-induced *TNF-α* expression was decreased by blocking MAPK or NF-κB signaling pathways. Phagocytic activity of RAW 264.7 cells was also significantly enhanced by neutral lipids and glycolipids. These results suggest that neutral lipids and glycolipids from *H. aurantium* tunic have potential as immune-enhancing materials.

## Introduction

*Halocynthia aurantium* (Pyuridae) is a marine ascidian primarily consumed in Korea and Japan. It can be found in the Eastern Sea of Korea, the Sea of Japan, and northern Russia [1-3]. Most of these species can be eaten raw, cooked, dried, or pickled. They are available in seafood markets [3]. In the genus *Halocynthia*, the morphology and composition of the tunic in different species like *H. aurantium*, *H. papillosa*, and *H. roretzi* have been thoroughly investigated [2, 4-6]. However, *H. aurantium* has received little research attention despite the fact that tunicates and ascidians are known to contain biologically active compounds [7-9].

Lipids are active constituents in marine ascidians. They play an essential role in modulating compositions of marine ascidians for health benefits [9, 10]. They are classified into two major classes based on their chemical characteristics, namely polar lipids (phospholipids, glycolipids, sphingolipids, etc.) and non-polar lipids also called neutral lipids such as triacylglycerol, cholesterol, wax, free fatty acids, etc. [11]. Polyunsaturated fatty acids (PUFAs) found in marine lipids, particularly eicosapentaenoic (EPA; 20:5 *n-3*) and docosahexaenoic (DHA; 22:6 *n-3*), are known to improve immune function [12]. Some studies have reported that total lipids, neutral lipids, and polar lipids from ascidian species contain high amounts of EPA and DHA [13-16]. Ascidian lipids possess health benefits such as anti-diabetic and antioxidant effects [17, 18].

*H. aurantium* has been reported to possess biological compounds such as antimicrobial peptides [19-21], antioxidants [22], gastroprotective [23], immune-enhancement [24] and anti-inflammatory activities [24]. Fatty acids extracted from *H. aurantium* tunic have been found to possess immune-enhancing and anti-inflammatory properties [24]. Other studies have shown that lipids and fatty acids derived from marine sources can promote several immune function effects in macrophages, resulting in immunological enhancement [25, 26]. Our previous study has revealed that total lipids isolated from *H. aurantium* tunic have immune regulatory effects and that neutral lipids, glycolipids, and phospholipids exhibit anti-inflammatory effects on RAW264.7 cells [18, 27]. They have high concentrations of PUFAs and other fatty acids [27]. The purpose of the present study was to evaluate immune-enhancing effects of neutral lipids, glycolipids, and phospholipids isolated from *H. aurantium* tunic on RAW264.7 cells along with their mechanisms.

## Materials and Methods

### Lipid Extraction and Fraction from *H. aurantium* Tunic

Total lipids were extracted from *H. aurantium* tunic using a modified method of Bligh and Dyer [28]. Briefly, lyophilized powder (4.5 g) of dried samples was mixed with a solution of chloroform and methanol (1:2, v/v) containing 0.01% of butylated hydroxytoluene (BHT) which served as an antioxidant [29] and centrifuged at 3000 rpm for 10 min. The homogenate was filtered and evaporated. After being dried, samples were resuspended in hexane.

To separate fractionated lipids, total lipids were added and separated by silica gel column chromatography on a glass column (250 mm × 1.8 mm) filled with silica gel (silica gel 60, Merck, Germany) and sodium sulfate (Samchun Chemical Co., Ltd., Republic of Korea). The column was eluted with 200 ml of chloroform as a solvent for neutral lipids, followed by 100 ml of acetone and 30 ml of methyl alcohol, which produced glycolipids and phospholipids, respectively. Solvents were evaporated with the rotary evaporator (IKA RV 10-digital, Germany) and nitrogen evaporator (12-position N-EVAP nitrogen evaporator, USA) to remove the solvent. The lipid content of neutral lipids, glycolipids, and phospholipids isolated from the total lipids was estimated as (%) of dry weight as described previously [27]. After evaporation, dried solvents were dissolved in dimethyl sulfoxide (DMSO, Sigma-Aldrich, USA) and stored at -20°C until analysis.

### Sample Treatments

RAW264.7 cells were purchased from from Koran Cell Line Bank (KCLB). These cells were grown at 37°C in RPMI-1640 medium (Gibco™, USA) supplemented with 10% fetal bovine serum (FBS, Welgene, Republic of Korea) and 1% penicillin/streptomycin (Welgene) in a humidified atmosphere of 5% CO_2_. In all experiments, each lipid was diluted with RPMI-1640 medium (no phenol red) supplemented with 1% FBS and 1% antibiotics before any treatments. Cells were pre-treated with various concentrations (0.5, 1.0, 2.0, and 4.0%) of three lipids or 1%DMSO as a control for 1 h. Following the addition of RPMI to wells, cells were further incubated for 24 h. The immune-enhancing effects of *H. aurantium* lipids were then evaluated.

### Cell Viability Analysis

RAW264.7 cells (1 × 10^6^ cells/ml) were tested for cytotoxicity using an EZ-Cytox Cell Viability Assay kit (DaeilLab Service, Republic of Korea). After removing the supernatant, treated cells were incubated at 37°C with the WST-solution for 1 h. Absorbance at 450 nm was then measure ith a microplate reader (BioTek Instruments, USA).

### Nitric Oxide (NO) Assay

After incubation for 24 h, nitric concentration in treated-lipid cells was measured using Griess reagent (Promega, USA). Culture supernatants were incubated with Greiss reagent A (1% sulfanilamide in 5% phosphoric acid) and Greiss reagent B (0.1% *N*-1-napthylethylenediamine dihydrochloride in water). Absorbance at 540 nm was then measured with a microplate reader (BioTek Instruments, USA).

### Measurement of Prostaglandin E2 (PGE_2_) Generation

Cells (1 × 10^6^ cells/ml) in a 24-well plate were pre-incubated with three lipids or 1% DMSO. Culture supernatants were collected at 24 h after incubation to determine PGE_2_ levels using ELISA kits (Enzo Life Sciences, USA) according to the manufacturer’s instructions.

### Real-Time PCR Analysis

To analyze relative expression levels of interleukin-1β (*IL-1β*), *IL-6*, tumor necrosis factor-α (*TNF-α*), cyclooxygenases-2 (*COX-2*), and *β-actin* in the immune system, qPCR was used as described previously [27]. After extracting total RNAs from cells with TRI reagent, cDNAs were synthesized from total RNAs with a high-capacity cDNA reverse transcription kit (Applied Biosystems, USA). For real-time PCR, cDNAs were added into a mixture of TB Green Premix Ex Taq II (Takara Bio Inc., Japan, Cat#RR820A) and oligonucleotide primers. PCR was then performed with a QuantStudio 7 FlexReal-Time PCR System (Applied Biosystems, USA).

### Western blotting Analysis

Cells were lysed with RIPA buffer (Tech & Innovation, China) containing 0.5 mM EDTA solution and a protease & phosphatase inhibitor cocktail (Thermo Fisher Scientific, USA) to release proteins. Proteins were then loaded and separated by SDS-polyacrylamide gel electrophoresis (SDS-PAGE) and transferred to a polyvinylidene fluoride (PVDF) membranes. Immunoblots were probed with primary antibodies against p-NF-κB p65, p-p38, p-ERK1/2, p-JNK (Cell Signaling Technology, USA) and α-tubulin (Abcam, UK), followed by incubation with secondary antibodies against goat anti-rabbit IgG (H+L)-HRP (GenDEPOT, USA). A Pierce ECL Plus Western Blotting Substrate (Thermo Fisher Scientific) was used to detect protein bands. Signal intensity was determined with a ChemiDoc XRS+ imaging system (Bio-Rad, USA).

### Pathway Inhibition Assay

RAW264.7 cells (1 × 10^6^ cells/ml) were treated with 100 nM of NF-κB activation inhibitor (Merck, USA) for 3 h and with 20 μM of ERK, JNK, and p38 activation inhibitors (Merck) for 1 h. The supernatant was removed and cells were treated with 4.0% of neutral lipids and glycolipids or 1 μg/ml of LPS. After 24 h, cells were extracted for total RNAs and gene expression levels were evaluated by real-time PCR. *TNF-α* expression was quantified using *β-actin* expression as a control.

### Measurement of Phagocytic Activity

RAW264.7 cells (1 × 10^6^ cells/ml) were treated with different concentrations of neutral lipids and glycolipids for 24 h. After incubation, cells were harvested and washed with cold PBS. Cells were then incubated with FITC-dextran (1 mg/ml) in RPMI-1640 at 37°C for 20 min in the dark. Cells were washed with PBS buffer, resuspended in 1% paraformaldehyde, and subjected to analysis of mean fluorescence intensity (MFI) using a CytoFLEX Flow Cytometer (Beckman Coulter, Inc., USA).

### Statistical Analysis

Statistix 8.1 Statistics Software (USA) was used to evaluate Statistical differences. Data were subjected to one-way analysis of variance (ANOVA) followed by Duncan's multiple range test with significance set at *p* < 0.05. Results are expressed as mean ± standard deviation (SD).

## Results

### Effects of Neutral Lipids, Glycolipids, and Phospholipids from *H. aurantium* Tunic on Cell Viability

**Fig. 1:**
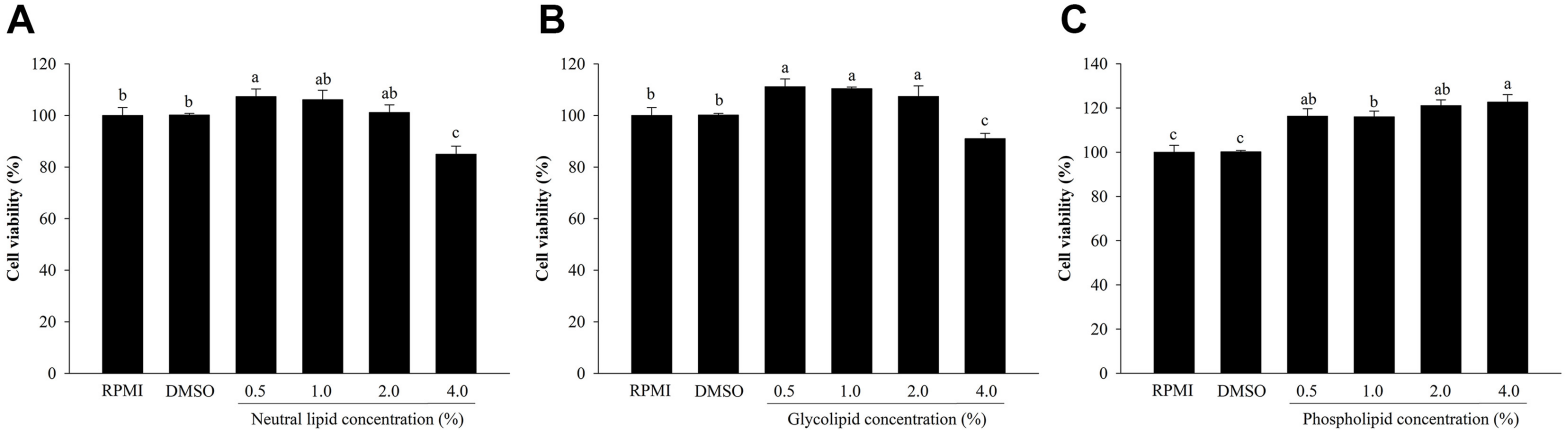
Effects of *H. aurantium* lipids on cell viability. (**A**) Neutral lipids, (**B**) glycolipids, and (**C**) phospholipids. Values are expressed as mean ± SD (*n* = 3). Different letters (a-c) indicate significant difference at *p* < 0.05.

Cytotoxicities of three lipids ot RAW264.7 macrophages were evaluated. Cell viability was not affected by neutral lipids or glycolipids at concentrations up to 2.0%. However, cell viability was reduced by approximately 84% after treatment with 4.0% of neutral lipids (Fig. 1A) and by 91% after treatment with 4.0% of glycolipids (Fig. 1B). As displayed in Fig. 1C, phospholipids had no effect on the viability of RAW264.7 cells.

### Effects of Neutral Lipids, Glycolipids, and Phospholipids from *H. aurantium* tunic on NO and PGE_2_ Production

**Fig. 2:**
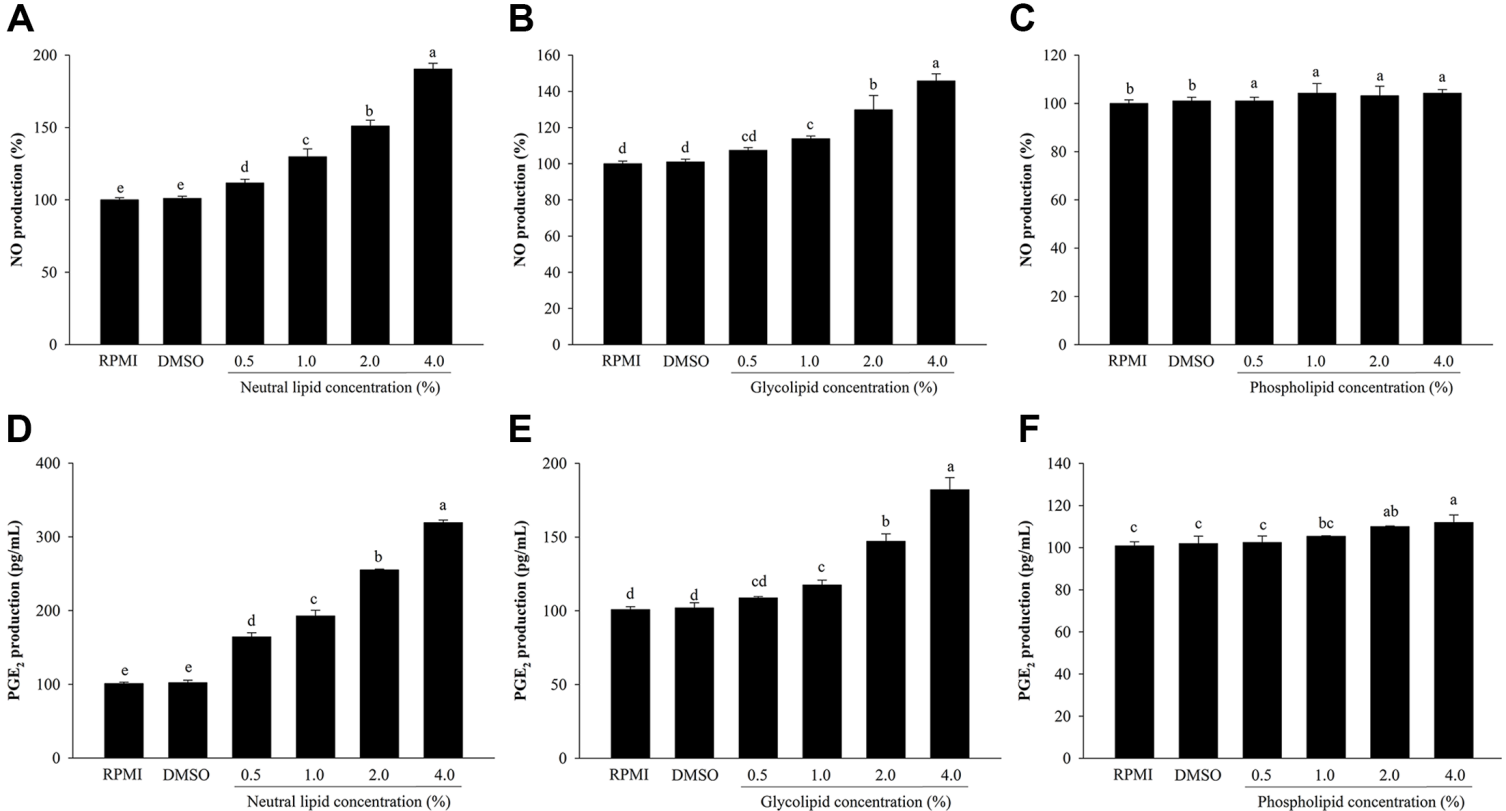
Effects of *H. aurantium* lipids on NO and PGE_2_ release. NO production levels in groups treated with neutral lipids (**A**) glycolipids (**B**) and phospholipids (**C**) are shown. PGE_2_ production levels in groups treated with neutral lipids (**D**), glycolipids (**E**), and phospholipids (**F**) are shown. Values are expressed as mean ± SD (*n* = 3). Different letters (a-e) indicate significant difference at *p* < 0.05.

To investigate effects of neutral lipids, glycolipids, and phospholipids on NO production, RAW264.7 cells were determined with Griess reagent. Results showed that treatment with 0.5–4.0% of neutral lipids or glycolipids significantly increased NO production in a dose-dependent manner compared to the control (Fig. 2A and 2B). In contrast, phospholipids did not significantly increase the production of NO compared to control (RPMI), although NO production was still increased by phospholipids in a dose-dependent manner (Fig. 2C).

Effects of three lipids on another inflammatory mediator, PGE_2_, were then investigated. As shown in Fig. 2D and 2E, neutral lipids and glycolipids dose-dependently increased PGE_2_ concentration. Such increases reached statistical significance after treatment with 4.0% of neutral lipids and glycolipids (by 319.24 and 182.09%, respectively). PGE_2_ concentrations in supernatants of cells treated with neutral lipids or glycolipids were higher than those in supernatants of control cells (RPMI and DMSO). However, phospholipids did not significantly increase PGE_2_ production at low concentrations. They only marginally increased PGE_2_ production at high concentrations, suggesting that phospholipids had no modulation effect on PGE_2_ production in cells (Fig. 2F).

### Effects of Neutral Lipids, Glycolipids, and Phospholipids from *H. aurantium* Tnic on Gene Expression

**Fig. 3:**
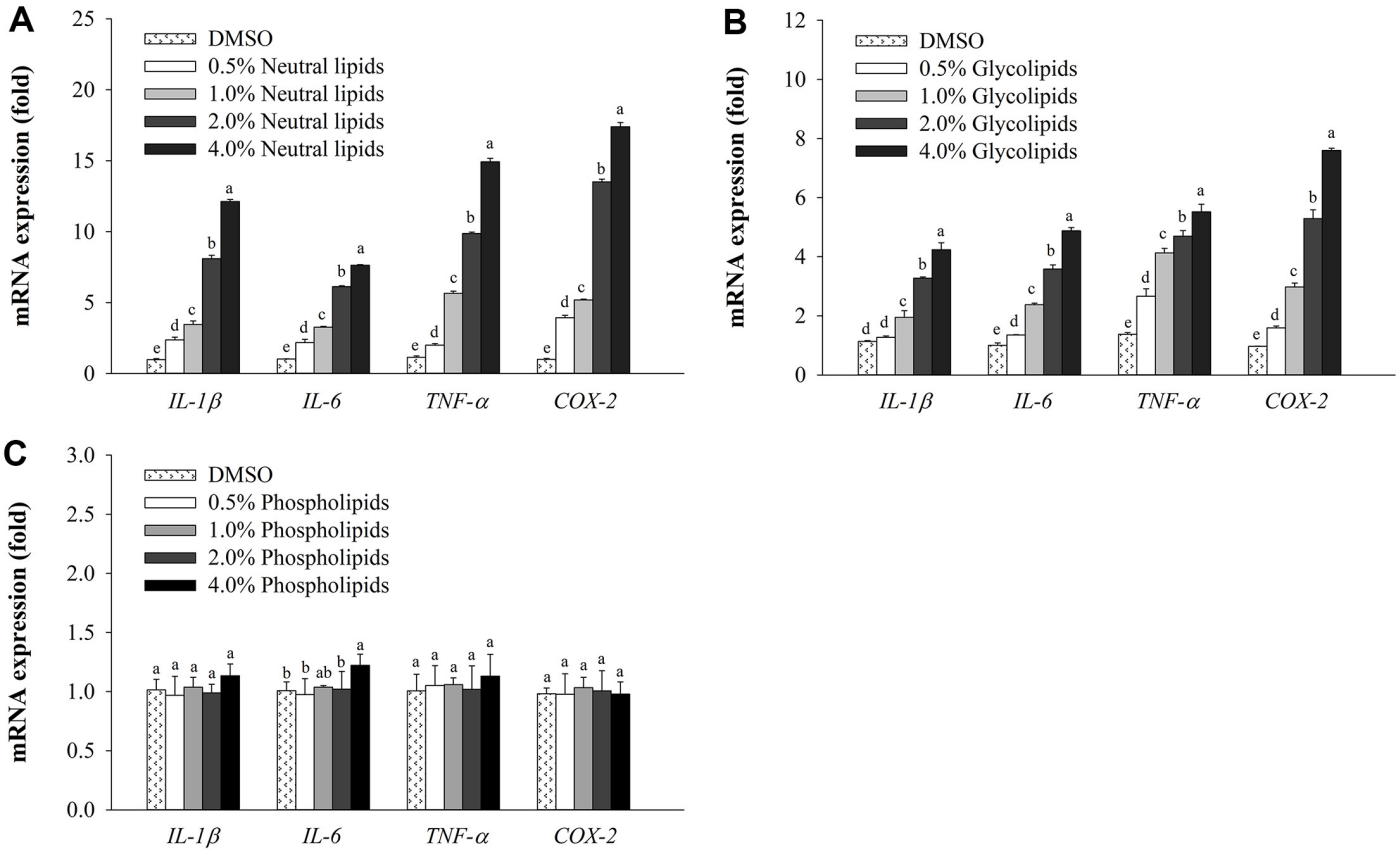
Effects of *H. aurantium* lipids on mRNA expression of immune genes. (**A**) Neutral lipids, (**B**) glycolipids, and (**C**) phospholipids. Values are expressed as mean ± SD (*n* = 3). Different letters (a-e) indicate significant difference at *p* < 0.05.

To determine whether *H. aurantium* lipids could enhance immunity in RAW264.7 cells, expression levels of immune-associated genes were measured. Except phospholipids, neutral lipids and glycolipids increased expression levels of cytokine genes such as *IL-1β*, *IL-6*, and *TNF-α* in a dose-dependent manner (Fig. 3A and 3B). In particular, *COX-2* expression was highly increased by neutral lipids and glycolipids. However, phospholipid did not show any effects on its expression (Fig. 3C). These data suggested that neutral lipids and glycolipids of *H. aurantium* might act as signaling molecules to increase pro-inflammatory cytokine secretion for immunological enhancement. Therefore, neutral lipids and glycolipids were selected for further experiments.

### Effects of Neutral Lipids and Glycolipids from *H. aurantium* Tunic on MAPK and NF-κB Signaling Pathways

**Fig. 4:**
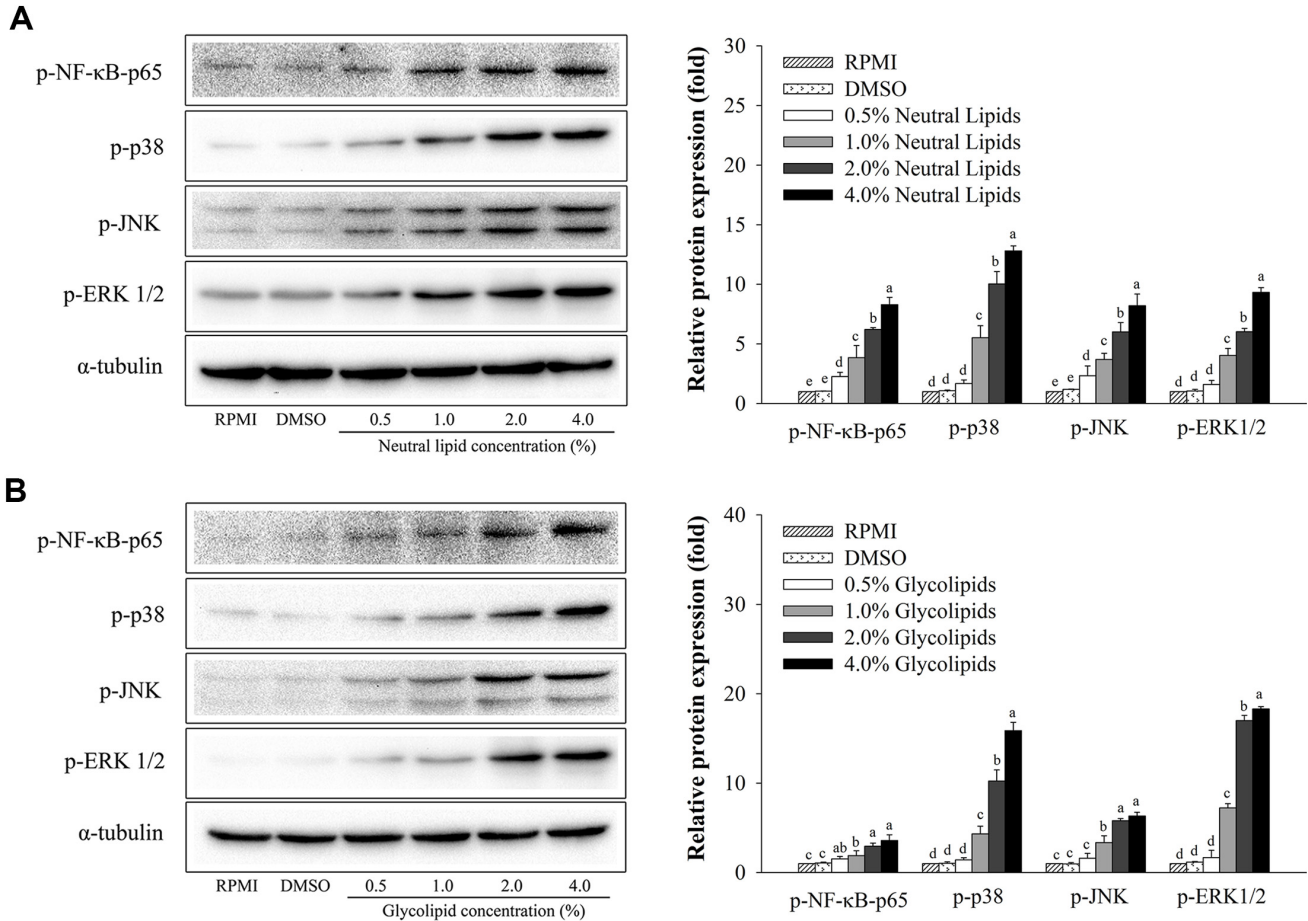
Effects of neutral lipids and glycolipids from *H. aurantium* tunic on expression levels of proteins associated with NF-κB and MAPK pathways. (**A**) Neutral lipids and (**B**) glycolipids. Values are expressed as mean ± SD (*n* = 3). Different letters (a-e) indicate significant difference at *p* < 0.05.

To determine phosphorylation levels of NF-κB and MAPK after treatment with *H. aurantium* lipids, western blot analysis was performed. As shown in Fig. 4, neutral lipids (Fig. 4A) and glycolipids (Fig. 4B) dose-dependently increased phosphorylation levels of NF-κB and MAPK. These results demonstrate that neutral lipids and glycolipids have immune-enhancing activity through NF-κB and MAPK signaling pathways in RAW264.7 macrophages.

### Effects of Neutral Lipids and Glycolipids from *H. aurantium* Tunic on MAPK and NF-κB Inhibited RAW264.7 Cells

**Fig. 5:**
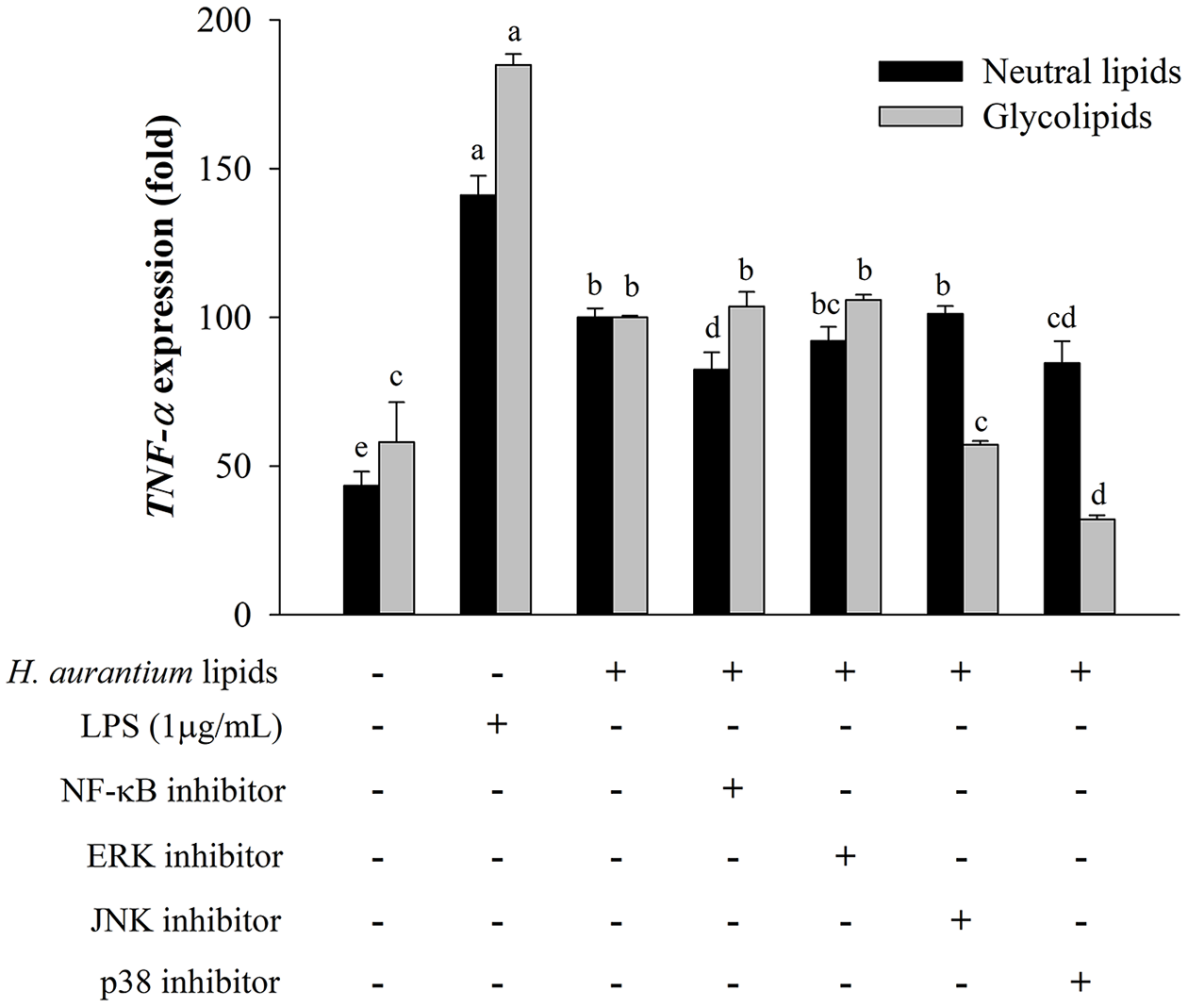
Effects of neutral lipids and glycolipids from *H. aurantium* tunic with specific NF-κB and MAPK inhibitors on *TNF-α* expression. Values are expressed as mean ± SD (*n* = 3). Different letters (a-e) indicate significant difference at *p* < 0.05.

To evaluate effects of neutral lipids and glycolipids on NF-κB and MAPK activation involved in the mechanism of immune-regulation, *TNF-α* expression was measured. After cells were pre-treated with different inhibitors of NF-κB, ERK, JNK, and p38, they were then incubated with 4.0% *H. aurantium* lipids for 24 h before being tested for *TNF-α* expression by real-time PCR. As shown in Fig. 5, *TNF-α* expression was increased in RAW264.7 cells treated with LPS as a positive control and *H. aurantium* lipids without inhibitors compared to RPMI, although *H. aurantium* lipids resulted in lower *TNF-α* expression than LPS. Compared to *H. aurantium* lipids without inhibitors, neutral lipids with specific NF-κB and p38 inhibitors suppressed *TNF-α* expression. JNK inhibitors had no effect on *TNF-α* expression, while ERK inhibitors had a statistically insignificant effect on *TNF-α* expression. Furthermore, *TNF-α* expression of glycolipids was reduced to levels similar to that in the group treated with glycolipids along with JNK and p38 inhibitors.

### Effects of Neutral Lipids and Glycolipids from *H. aurantium* Tunic on Phagocytic Ability

**Fig. 6:**
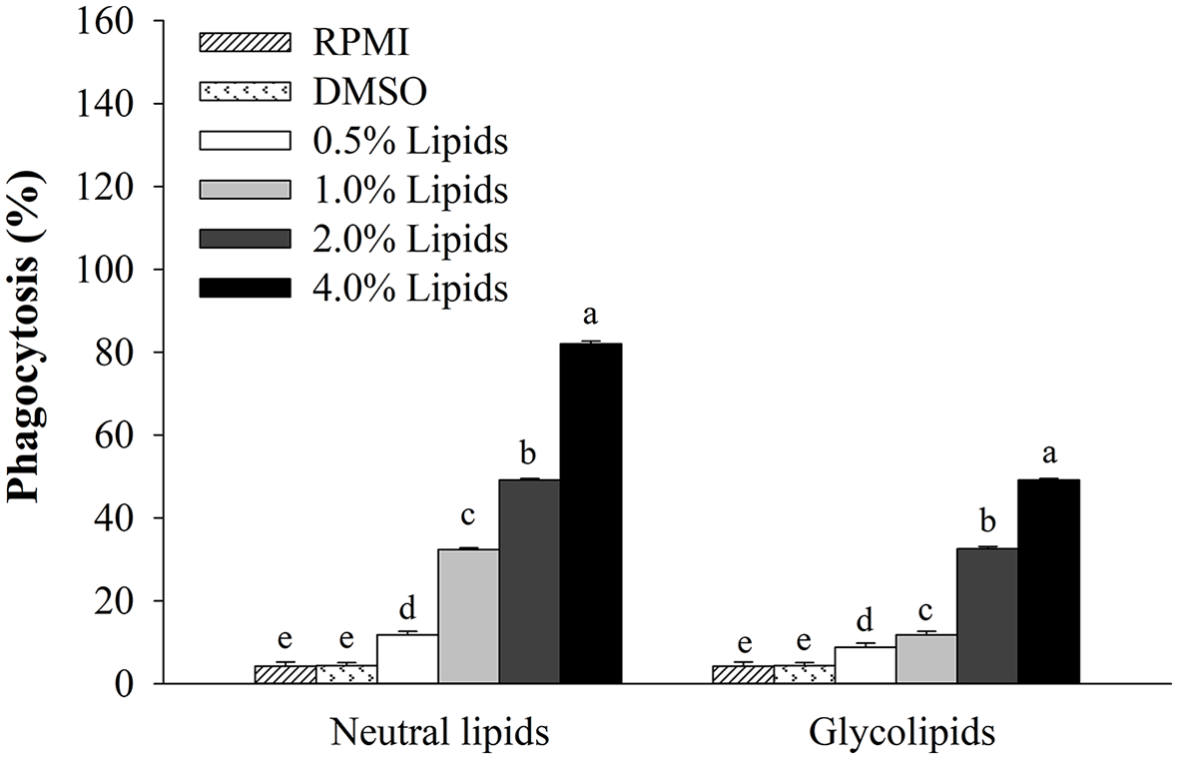
Effects of neutral lipids and glycolipids from *H. aurantium* tunic on macrophage phagocytosis. Values are expressed as mean ± SD (*n* = 3). Different letters (**a-e**) indicate significant difference at *p* < 0.05.

In a subsequent study, FITC-dextran uptake by macrophages was assessed in the presence of neutral lipids, glycolipids, or DMSO. As shown in Fig. 6, the phagocytic activity of RAW264.7 cells was elevated after 24 h of incubation with neutral lipids or glycolipids. Both lipids boosted the phagocytic activity of RAW264.7 cells, which displayed higher FITC-dextran uptake than the control group. Neutral lipids at 0.5%, 1.0%, 2.0%, and 4.0%significantly increased phagocytosis by 11.89%, 32.37%, 49.10%, and 82.04%, respectively. Glycolipids at 0.5%, 1.0%, 2.0%, and 4.0% also increased FITC-dextran uptake by 8.77%, 11.81%, 32.53%, and 49.10%, respectively.

## Discussion

Three lipids, including neutral lipids, glycolipids, and phospholipids, were isolated from total lipids of *H. aurantium* tunic. Our previous study has demonstrated that these lipids show anti-inflammatory properties and contain diverse essential fatty acids [27]. In the present study, these lipids were evaluated for their immune-enhancing effects on macrophage cells.

Several studies have demonstrated immunomodulatory activities of bioactive compounds that can enhance the production of reactive oxygen species (ROS) and NO as well as the production of cytokines and chemokines such as IL-1β, IL-6, IL-12, IL-10, TNF-α, and TGF-β in RAW264.7 macrophages [25, 30-32]. Fatty acids from *H. aurantium* tunic have been reported to possess immunomodulatory properties. These compounds could regulate the production of NO and PGE_2_ and the expression of *iNOS*, *IL-1β*, *IL-6*, *COX-2*, and *TNF-α* via MAPK and NF-κB signaling [24]. Han *et al*. (2018) have demonstrated that DHA can stimulate GPR120, C-Raf, and MAPKs to activate the NF-κB p65 pathway, which increases mRNA and protein expression of iNOS as well as cytokine production of IL-1β, IL-6, IL-10, IL-12, TNF-α, IFN-γ, and TGF-β [25]. Results of the present study showed that neutral lipids and glycolipids significantly increased the production of NO and PGE_2_ in RAW264.7 macrophages compared to the control group. In addition, expression levels of immune-associated genes such as *IL-1β*, *IL-6*, and *TNF-α* as well as *COX-2* were increased by neutral lipids and glycolipids in a dose-dependent manner (Fig. 3). Cyclooxygenases are metabolic enzymes involved in the production of prostaglandins, which are important components of inflammatory responses [33]. Lipids derived from *Ammodytes personatus* (Ammodytidae) and *H. aurantium* can increase the expression of *COX-2* which can stimulate the production of PGE_2_ [26]. Especially, our study showed that phospholipids did not stimulate NO production or gene expression. However, neutral lipids and glycolipids increased NO production and immune gene expression, suggesting that these two lipids were involved in the immunostimulatory activity of *H. aurantium* lipids. One of the critical mechanisms for improving immune function is the ability to regulate macrophage phagocytosis [34], which is consistent with immunomodulators provided by many natural compounds in RAW264.7 macrophages [34-37]. Our results demonstrate that neutral lipids and glycolipids could activate macrophages to increase their phagocytic activities.

Additionally, effects of neutral lipids and glycolipids isolated from *H. aurantium* on NF-κB and MAPK signaling pathways were evaluated. Our results showed that these two lipids enhanced protein expression levels, leading to further activation of phosphorylated NF-κB p-65 and phosphorylated MAPK molecules such as ERK1/ 2, JNK, and p38 (Fig. 4). Moreover, *TNF-α* expression caused by NF-κB and MAPK inhibition was differently regulated when NF-κB and MAPK signaling were inhibited, meaning that neutral lipids and glycolipids enhanced immunity with different signaling pathways in macrophages (Fig. 5). Alfalfa (Fabaceae) polysaccharides can reduce TNF-α production by blocking NF-κB or MAPK inhibitor in RAW 264.7 macrophages [31], similar to our results.

## Conclusion

Results of the current study demonstrated that neutral lipids and glycolipids isolated from *H. aurantium* tunic stimulated inflammatory mediator production and immune-associated gene expression in RAW264.7 cells. Expression levels of phosphorylated NF-κB p-65, ERK1/2, JNK, and p38 were enhanced by neutral lipids and glycolipids. On the other hand, phospholipids did not stimulate macrophage function. These results indicate that neutral lipids and glycolipids in *H. aurantium* tunic exhibit potential immune-enhancing activities.
